# Early Laboratory Predictors for Necessity of Renal Replacement Therapy in Patients With Spontaneous Deep-Seated Intracerebral Hemorrhage

**DOI:** 10.3389/fneur.2021.636711

**Published:** 2021-02-25

**Authors:** Lorena M. Schenk, Matthias Schneider, Christian Bode, Erdem Güresir, Christoph Junghanns, Marcus Müller, Christian Putensen, Hartmut Vatter, Julian Zimmermann, Patrick Schuss, Felix Lehmann

**Affiliations:** ^1^Department of Neurosurgery, University Hospital Bonn, Bonn, Germany; ^2^Department of Anesthesiology and Critical Care Medicine, University Hospital Bonn, Bonn, Germany; ^3^Department of Neurology, University Hospital Bonn, Bonn, Germany

**Keywords:** intracerebral hemorrhage, renal replacement therapy, acute kidney injury, procalcitonin, critical care (ICU)

## Abstract

**Objective:** The need for continuous renal replacement therapy (CRRT) in patients with deep-seated intracerebral hemorrhage (ICH) requires sustained intensive care and often postpones further rehabilitation therapy. Therefore, an early identification of patients at risk is essential.

**Methods:** From 2014 to 2019, all patients with deep-seated ICH who were admitted to intensive care for >3 days were included in the further analysis and retrospectively reviewed for the need for CRRT. All patients underwent CRRT with regional citrate anticoagulation for continuous veno-venous hemodialysis (CVVHD). Outcome was evaluated after 3 months using the modified Rankin scale. A multivariate analysis was performed to identify potential predictors for CRRT in patients with deep-seated ICH.

**Results:** After applying the inclusion criteria, a total of 87 patients with deep-seated spontaneous ICH were identified and further analyzed. During the first 48 h after admission, 21 of these patients developed early acute kidney injury (AKI; 24%). During treatment course, CRRT became necessary in nine patients suffering from deep-seated ICH (10%). The multivariate analysis revealed “development of AKI during the first 48 h” [*p* = 0.025, odds ratio (OR) 6.1, 95% confidence interval (CI) 1.3–29.8] and “admission procalcitonin (PCT) value >0.5 μg/l” (*p* = 0.02, OR 7.7, 95% CI 1.4–43.3) as independent and significant predictors for CRRT in patients with deep-seated ICH.

**Conclusions:** Elevated serum levels of procalcitonin on admission as well as early development of acute renal injury are independent predictors of the need for renal replacement therapy in patients with deep-seated intracerebral bleeding. Therefore, further research is warranted to identify these vulnerable patients as early as possible to enable adequate treatment.

## Introduction

Acute renal injury (AKI) is a frequent and devastating complication with high morbidity and mortality in patients requiring treatment in an intensive care unit (ICU). Previous studies have reported an incidence of AKI up to 67% depending on the definition of AKI and the underlying cause of ICU admission (1, 2). With regard to neurological diseases, the presence of chronic kidney disease (CKD) in patients with acute stroke (ischemic/hemorrhagic) was identified as a strong independent predictor for both mortality and adverse outcomes (3). Both the increased appearance of atherosclerotic alterations and a less effective dynamic cerebral autoregulation in acute stroke were discussed as potential explanations for this correlation (3, 4). Furthermore, mortality increases dramatically with growing severity of AKI, resulting in patients with the need for continuous renal replacement therapy (CRRT) accounting for the highest mortality (1).

However, much of the data on the kidney–brain interaction focuses on patients with ischemic or undifferentiated stroke (5). Intracerebral hemorrhage (ICH) constitutes a major hemorrhagic manifestation of acute stroke (6). Regarding ICH, further evaluation of the INTERACT2 data has revealed a prognostic value for decreased estimated glomerular filtration rate (eGFR) at admission (5). Initial lowering of systolic blood pressure is an important therapeutic intervention in the treatment of ICH to prevent further hematoma expansion (7). Especially in border-compensated patients, deterioration of renal function in the acute situation (e.g., due to aggressive blood pressure management) might accelerate the development of AKI (8). In addition, the need for CRRT in patients with ICH requires continued intensive care treatment and often results in further postponement of a potential rehabilitation therapy. This highlights the need for early identification and treatment of these particularly endangered patients.

Therefore, the aim of the present study was to investigate both the incidence and the influence of needed CRRT on mortality in a selected cohort of neurocritically ill patients with deep-seated ICH. Furthermore, we attempted to identify risk factors for the necessity of CRRT in this specific subpopulation of critically ill patients with ICH.

## Materials and Methods

### Patients

Medical records of patients treated for deep-seated spontaneous intracerebral hemorrhage between 2014 and 2019 at the Neurosurgical Department of the University Hospital Bonn, Germany, were retrospectively reviewed. Patients were identified using the ICD coding system and verified as eligible for study inclusion by three authors (LMS, PS, and FL). Hemorrhages originating from the area of the basal ganglia and/or thalamus were classified as deep-seated ICH. All patients with supratentorial deep-seated ICH who developed AKI with or without CRRT during the course of treatment were included in the further analysis after approval of the local IRB. Patients with lobar ICH and/or ICH with underlying bleeding source (e.g., aneurysm, arteriovenous malformation, and trauma) were excluded from this study. In addition, all patients in whom no further treatment or treatment for <3 days in intensive care was initiated due to the devastating clinical situation and/or an existing patient wish for withdrawal of life-sustaining treatment were excluded from further analysis. Information collected for each patient included general characteristics, ICH location, ICH volume (9), ICH score (10), parameters of intensive care and laboratory, necessity of surgical intervention, need for hyperosmolar therapy, occurrence of AKI, necessity of CRRT, neurological status at admission, 3-month outcome/mortality, and treatment strategies during hospitalization. Initial systolic blood pressure (SBP) was categorized into mild (<180 mmHg), moderate (180–219 mmHg), and severe (≥220 mmHg), as previously described (11). In addition, all patients with infratentorial localization of ICH were excluded from further analysis. In the case of extensive space-occupying hemorrhage, the affected patients were assigned by two authors (LMS and FL) to either the lobar or deep-seated group, depending on probability and image morphologic findings. If any disagreement occurred regarding the classification between these two authors, it was resolved in a consensus meeting with the senior neurosurgical author (PS).

All patients suffering from deep-seated ICH received the best medical treatment according to the hospital's in-house standard operating procedures, which comply with the guidelines of the American Heart Association/American Stroke Association (12).

Modified Rankin scale (mRS) was applied to assess functional outcome. Patients were dichotomized according to mRS into two groups: (1) favorable outcome (mRS 0–4) versus (vs.) (2) unfavorable outcome (mRS 5–6), as defined in previous studies (13, 14).

In order to assess renal function, the daily serum creatinine (SCr) was assessed over the first 3 days after admission as defined in a previous study (8). Early onset of AKI was defined and graded according to the KDIGO (Kidney Disease: Improving Global Outcomes) guidelines: a minimum increase in SCr of either ≥0.3 mg/dl or >150% of baseline SCr during the first 48 h after admission (15). Urinary output was not considered for definition or staging of the AKI in the present study due to limited data. The decision to initiate CRRT was made by intensive care physician/neurosurgeon according to the current international and national guidelines (16–18). CRRT was performed using regional citrate anticoagulation for continuous veno-venous hemodialysis (CVVHD) in all patients included in the present analysis. All patients were observed for at least 3 months and were divided into two groups according to the presence or absence of CRRT for further analysis.

### Statistics

Data analyses were performed using the computer software package SPSS (version 25, IBM Corp., Armonk, NY). Mann–Whitney test was used for nonparametric statistics after testing for normal distribution. Categorical variables were analyzed in contingency tables using Fisher's exact test. Results with *p* < 0.05 were considered statistically significant. In addition, in order to determine independent predictors of the necessity of CRRT during treatment course in patients with deep-seated ICH, a multivariate analysis using binary logistic regression was performed. Variables with significant *p*-values in the univariate analysis, as well as variables that were considered meaningful in the clinical context, were considered potentially independent variables in a multivariate analysis. A backward stepwise method was used to construct a multivariate logistic regression model in relation to the CRRT as a dependent variable with an inclusion criterion of a *p*-value < 0.05.

## Results

### Patient Characteristics

Overall, 87 patients suffering from deep-seated spontaneous ICH were identified and further analyzed. Initial SBP was mild (<180 mmHg) in 61 patients (65%), moderate (180–219 mmHg) in 23 patients (25%), and severe (≥220 mmHg) in 10 patients (10%). During the first 48 h after admission, 21 of these patients developed early AKI (24%). Of these patients, 18 patients had early AKI stage 1 (86%), two patients with early AKI stage 2 (10%), and one patient with early AKI stage 3 (4%). During treatment course, CRRT became necessary in nine patients suffering from deep-seated ICH (10%). CRRT was required on median day 9 (range 1–22) after admission. Neither required surgical intervention due to the space-occupying effect of deep-seated ICH nor the administration of hyperosmolar therapy as part of intracranial pressure treatment was found to have a significant effect on the need for CRRT (*p* = 0.73; *p* = 0.16). Further baseline characteristics of the present study cohort are given in Table 1.

**Table 1:** Baseline patient characteristics.

	**Non-CRRT (*n* = 78)**	**CRRT (*n* = 9)**	
Age (mean ± SD, years)	64 ± 14	60 ± 11	*p* = 0.41
Female gender	27 (35%)	3 (33%)	*p* = 0.63
GCS >12 at admission	28 (36%)	2 (22%)	*p* = 0.33
GCS <8 at admission	24 (31%)	4 (44%)	*p* = 0.32
ICH volume (abc/2, mean ± SD)	47.1 ± 42.3	54.5 ± 33.6	*p* = 0.61
Presence of IVH	48 (61%)	7 (78%)	*p* = 0.29
ICH score ≥3 at admission	32 (41%)	6 (67%)	*p* = 0.13
**Initial SBP (mmHg)**			
Mild (<180)	51 (65%)	6 (67%)	*p* = 1.0
Moderate (180–219)	18 (23%)	2 (22%)	*p* = 1.0
Severe (≥220)	9 (12%)	1 (11%)	*p* = 1.0
Hyperosmolar therapy	9 (12%)	1 (11%)	*p* = 0.73
Early AKI (≤48 h after admission)	15 (19%)	6 (67%)	*p* = 0.005, OR 8.4, 95% CI 1.9–37.5
Surgical treatment through treatment course	18 (23%)	4 (44%)	*p* = 0.16
Baseline SOFA score (mean ± SD)	4 ± 3	6 ± 2	*p* = 0.072
Baseline SAPS score (mean ± SD)	40 ± 17	48 ± 13	*p* = 0.15
Baseline SCr (mean ± SD, mg/dl)	0.99 ± 0.78	1.60 ± 0.77	*p* = 0.03, 95% CI 0.07–1.2
Baseline CRP (mean ± SD, mg/l)	15.7 ± 33.9	31.9 ± 55.2	*p* = 0.21
Baseline PCT (mean ± SD, μg/l)	0.20 ± 0.45	1.11 ± 1.79	*p* = 0.0004, 95% CI 0.4–1.4
Baseline WBC (mean ± SD, g/l)	11.06 ± 4.40	13.26 ± 7.09	*p* = 0.19
Baseline Glc (mean ± SD, mg/dl)	146 ± 69	151 ± 63	*p* = 0.84
Length of hospital stay (days)	20 ± 16	73 ± 79	*p* < 0.0001, 95% CI 33.7–72.3
90-day mortality	30 (39%)	7 (78%)	*p* = 0.03, OR 5.6, 95% CI 1.09–28.8

*CRRT, continuous renal replacement therapy; SD, standard deviation; GCS, Glasgow Coma Scale; ICH, intracerebral hemorrhage; IVH, intraventricular hemorrhage; SBP, systolic blood pressure; AKI, acute kidney injury; SOFA, sepsis-related organ failure; SAPS, simplified acute physiology; SCr, serum creatinine; CRP, c-reactive protein; PCT, procalcitonin; WBC, white blood cells; Glc, glucose; mRS, modified Rankin scale*.

### Influence of Admission Laboratory Results

**Figure 1:**
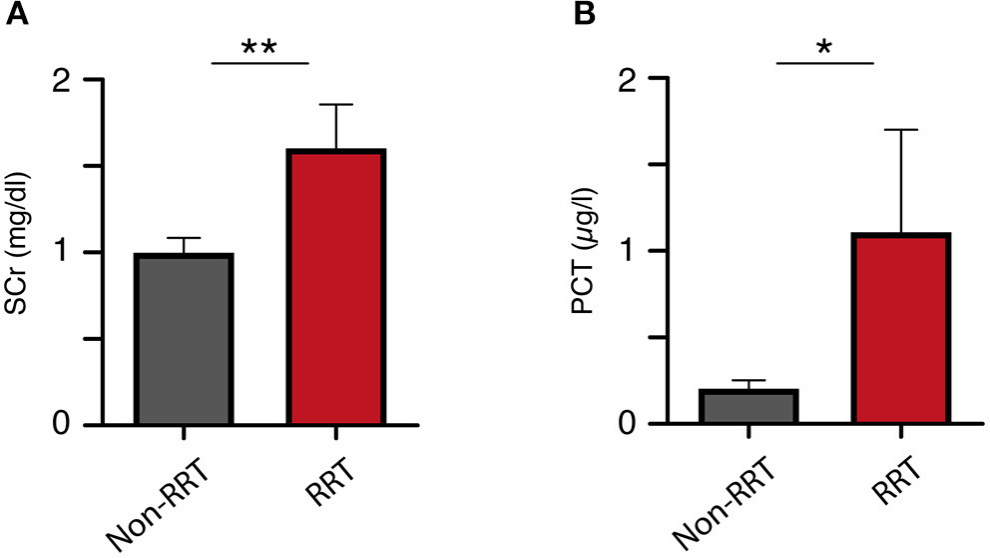
Initial **(A)** serum creatinine and **(B)** procalcitonin levels correlate to the rate of renal replacement therapy. *p = 0.024; **p = 0.009.

Mean serum concentrations of creatinine as well as procalcitonin (PCT) were significantly higher in the group with subsequent CRRT at the time of ICU admission (Table 1). ICH patients without CRRT during treatment course presented with a mean admission SCr concentration of 0.99 ± 0.78 mg/dl compared to 1.60 ± 0.77 in ICH patients with subsequent CRRT (*p* = 0.009; Figure 1A). Furthermore, patients with CRRT during treatment course presented with a significantly higher mean PCT concentration (1.11 ± 1.79 μg/l) compared to patients without CRRT [0.20 ± 0.45 μg/l; *p* = 0.024 95% confidence interval (CI) 0.4–1.4; Figure 1B]. Mean CRP laboratory value, mean WBC count, as well as mean glucose concentration at admission did not differ significantly between patients with and without CRRT.

### Outcome

**Figure 2:**
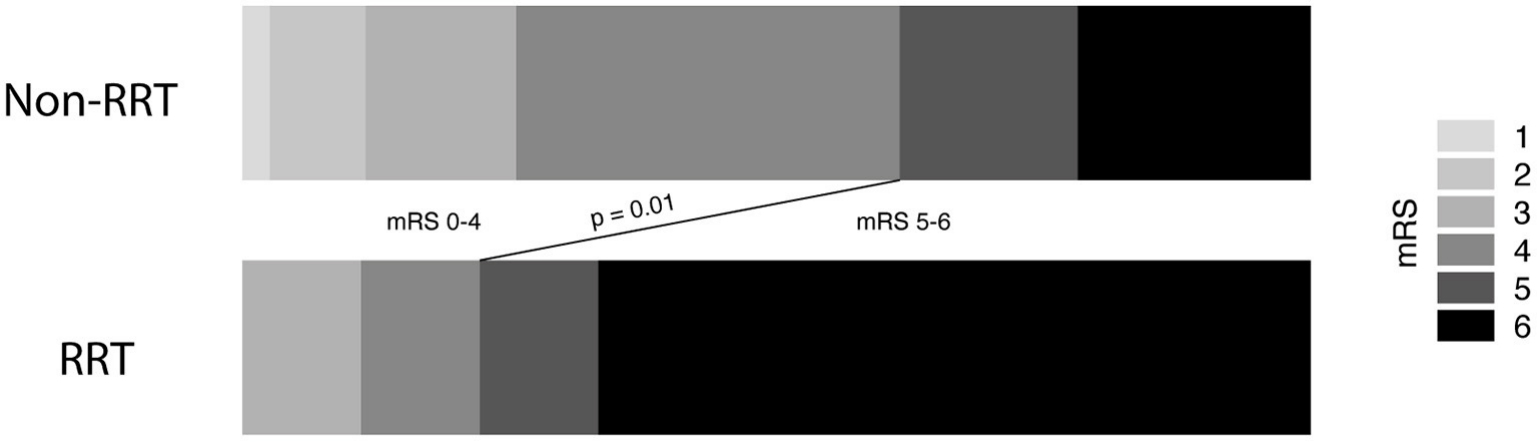
Necessity of renal replacement therapy correlates to unfavorable outcome in intracerebral hemorrhage disease.

Overall, mortality rate after 3 months was 43%. Mortality rates differed significantly between patients with and without CRRT (78 vs. 39%; *p* = 0.03). Patients with deep-seated ICH and subsequent CRRT remained significantly longer hospitalized compared to ICH patients without CRRT (*p* < 0.0001, 95% CI 33.7–72.3). In addition, patients with deep-seated ICH and subsequent CRRT achieved significantly less often favorable outcome assessed at the 3 months follow-up examination compared to patients without the necessity of CRRT [22 vs. 62%; *p* = 0.03, odds ratio (OR) 5.6, 95% CI 1.09–28.8; Table 1, Figure 2].

### Multivariate Analysis

We performed a multivariate regression analysis to identify independent predictors for the necessity of CRRT during the treatment course of patients suffering from deep-seated ICH. Herein, “development of AKI during the first 48 h” (*p* = 0.025, OR 6.1, 95% CI 1.3–29.8) and “admission PCT value >0.5 μg/l” (*p* = 0.02, OR 7.7, 95% CI 1.4–43.3) were identified as the only independent and significant predictors for CRRT in patients with deep-seated ICH (Nagelkerke's *R*^2^ = 0.295; Table 2).

**Table 2:** Multivariate logistic regression analysis of independent factors related to necessity of renal replacement therapy in patients with deep-seated ICH.

**Factors**	**Adjusted OR**	**95% CI**	***p*-value**
Age ≥65 years	2.3	0.3–20.7	0.4
Presence of IVH	0.7	0.09–4.8	0.7
Hyperosmolar therapy	0.8	0.06–11.9	0.9
**Early AKI (within 48 h)**	**6.1**	**1.3**–**29.8**	**0.025**
Baseline CRP >3 mg/l	2.5	0.2–26.6	0.5
**Baseline PCT** **>0.5** **μg/l**	**7.7**	**1.4**–**43.3**	**0.02**
Baseline SCr >1.2 mg/dl	0.4	0.04–2.9	0.3
Baseline WBC >12 g/l	0.3	0.05–1.6	0.1

*ICH, intracranial hemorrhage; OR, odds ratio; CI, confidence interval; IVH, intraventricular hemorrhage; AKI, acute kidney injury; CRP, C-reactive protein; PCT, procalcitonin; SCr, serum creatinine; WBC, white blood cell. The significant values of the multivariate analysis are shown in bold*.

## Discussion

In general, AKI occurs in approximately 10–15% of patients admitted to the hospital, whereas its incidence in the ICU has been reported in more than 50% of patients (19). In neurological patients, AKI appears to be somewhat less common. In a *post-hoc* analysis of pooled data from randomized clinical trials of acute ischemic stroke, AKI was diagnosed in 3.5% of patients (20). The present study reveals a considerable prevalence of early AKI in patients with deep-seated ICH, namely, 24%. Furthermore, this single-center series provides descriptive analysis and identifies predictors regarding the need for CRRT, which are easily available at an early stage in the treatment of patients with deep-seated ICH. The present multivariate regression analysis indicates that an increased baseline PCT laboratory value at time of admission and the development of AKI during the first 48 h of hospital treatment are independent and significant predictors of the development of renal failure with subsequent CRRT over the course of treatment in patients with deep-seated ICH.

Acute renal failure often occurs during the treatment of critically ill patients who subsequently require some form of RRT. However, patients with ICH represent a distinct patient population that merits special attention when planning RRT (21). The main focus of treatment is the prevention of secondary brain damage by maintaining adequate cerebral blood flow (CBF) by controlling cerebral perfusion pressure (CPP) and intracranial pressure (ICP). These treatment goals could be seriously affected by RRT. Thus, patients with ICH are extremely sensitive to osmotic gradients/shifts, and even minor changes might lead to exacerbation of brain edema/ICP. In the present study, patients with deep-seated ICH and CRRT achieved significantly worse functional outcome. In addition to the criticism regarding the definition of favorable outcome in studies investigating destructive pathologies in high-eloquent cerebral areas, patients requiring CRRT are considerably longer confined to an appropriate intensive care unit. Furthermore, immobilization is likely to result from intensified apparatus medicine (herein: CRRT) in addition to the initial impairment due to hemorrhage (22).

A previous meta-analysis revealed that AKI is a frequent complication with a prevalence of 19% in patients with ICH, although the nonsignificant influence of AKI on mortality in ICH may be due to lack of studies on this topic (23). The authors included only two studies reporting prevalence and mortality of AKI in patients after ICH. Many clinical studies investigating ICH seem to exclude patients with AKI due to its identification as a potential outcome modifier (23).

In the present study, increased serum procalcitonin at the time of admission was an additional significant predictor of the necessity of CRRT during the course of treatment in patients with deep-seated ICH (*p* = 0.02). PCT is a widely available specific biomarker for bacterial infections and has additional benefits that make it a serially used diagnostic marker in intensive care medicine (24, 25). While much has been reported on PCT tests for diagnosing infectious diseases, relatively little attention has been paid to its potential role in the diagnosis and treatment of non-infectious diseases (26). Nevertheless, a recent retrospective study found a significant association between increased serum PCT at hospital admission and the subsequent development of AKI in critically ill, non-septic patients (27). This association could be supported by the assumption that a decrease in renal function (whether acute or chronic) might lead to increased serum concentrations of proinflammatory metabolites (28). Thus, the latter would stimulate the immune system, which in turn could result in an aggravated inflammatory response and thus an increased release of PCT into the circulation (28, 29). Regarding ICH, a prospective cohort study in patients with primary ICH indicated an association between serum PCT levels and clinical outcome (30). Another study on the influence of elevated PCT levels in patients with clinically and radiologically severe aneurysmatic subarachnoid hemorrhage assumed that these patients suffered more severely from cerebral circulatory disturbance at the time of bleeding (31). Thus, the PCT values probably reflect an acute systemic stress response to the bleeding (31). Another consideration might be an early depiction of aspiration pneumonia in comatose patients with ICH given the elevated PCT levels at admission (32). An important finding regarding the applicability of PCT beyond the identification of infections is that PCT values are higher in patients with impaired renal function and that hemodialysis values can decrease by up to 80% (26, 29).

### Limitations

The present study has several limitations. Statistical analysis and data collection were done retrospectively, of which the available data represent only a single-center experience. Furthermore, only patients with deep-seated ICH as well as patients who were in stationary care for a certain period of time were assessed and further analyzed. This might result in a significant level of selection bias. However, this high level of selection is also considered a strength of the present study, as certain influencing factors (underlying pathologies, therapy limitation due to patient desire/disastrous condition) can therefore be excluded. Nevertheless, future studies should focus even more on developing advanced predictive models for the forecast of these intensive care complications in patients with ICH to enable treating physicians to further optimize/adapt the treatment and counseling of patients/family members.

## Conclusions

The present study identifies elevated serum levels of procalcitonin at admission, as well as an early development of acute kidney injury, as independent predictors of the necessity of renal replacement therapy in patients with deep-seated intracerebral bleeding. Therefore, further research is warranted to identify these critically ill and additionally endangered patients as early as possible in order to provide adequate treatment.

## Data Availability Statement

The raw data supporting the conclusions of this article will be made available by the authors, without undue reservation.

## Ethics Statement

The studies involving human participants were reviewed and approved by Ethics committee, University Hospital Bonn. Written informed consent for participation was not required for this study in accordance with the national legislation and the institutional requirements.

## Author Contributions

PS and FL conceptualized the study. MS, PS, and FL were responsible for the methodology. LS, MS, PS, and FL performed data collection. LS, PS, and FL were responsible for the statistics and wrote the original draft. MS and PS were responsible for the figures. PS and FL supervised the study. All authors contributed to the article and approved the submitted version.

## Conflict of Interest

The authors declare that the research was conducted in the absence of any commercial or financial relationships that could be construed as a potential conflict of interest.
